# Identification of insulin and glucagon genes in the finless porpoise and the developmental distribution of their producing endocrine cells

**DOI:** 10.3389/fendo.2026.1777351

**Published:** 2026-04-30

**Authors:** Liyuan Zhao, Likun Wang, Reyilamu Aierken, Qingfan Zeng, Yan Tan, Yuke Zhang, Qianhui Zeng, Xianyan Wang, Mingyu Li

**Affiliations:** 1 Fujian Provincial Key Laboratory of Marine Ecological Conservation and Restoration, Ministry of Natural Resources, Xiamen, China; 2 Fujian Provincial Key Laboratory of Innovative Drug Target Research, School of Pharmaceutical Sciences, Xiamen University, Xiamen, China

**Keywords:** endocrine cells, finless porpoises, glucagon, insulin, pancreatic islet

## Abstract

**Background:**

Both insulin and glucagon have been extensively studied due to their critical roles in glucose metabolism, development, and disease. However, there is limited information about the molecular characteristics of insulin and glucagon in cetaceans.

**Methods:**

To better understand the information of these key endocrine factors of finless porpoises, we cloned and characterized both of the *preproinsulin* and *preproglucagon* genes and determined the islet distribution, architecture, and composition from different growth stages in finless porpoises.

**Results:**

The coding sequences of the *preproinsulin* and *preproglucagon* genes encode the preproinsulin protein of 110 amino acid (aa) residues and the preproglucagon protein of 180 aa residues, respectively. Both of the *insulin* and *glucagon* genes exhibited strong conservation in primary structure and synteny to other mammals. The islet area and composition in different stages or gender of finless porpoises were different. The α cells and β cells started to form an islet in the late fetus stage and appeared to be randomly distributed under incompletely mature status. In the pregnant stage, the islets displayed β cells in the islet core, with most α cells in the islet periphery. Interestingly, the area of β cells increased in pregnant female porpoises.

**Conclusion:**

These findings provide new insights into the structural conservation of insulin and glucagon genes as well as the developmental distribution of endocrine cells in cetaceans, which may also be useful to their conservation, such as in assessing endocrine-disrupting chemicals’ risks in the future.

## 1 Introduction

Insulin and glucagon are master regulators of glucose homeostasis. Insulin is secreted by pancreatic β cells in response to elevated glucose. It stimulates peripheral tissues in its uptake of glucose and maintains normal blood glucose levels (1). Glucagon is produced by pancreatic α cell and plays the opposite actions of insulin on glucose metabolism. In the fasting state, glucagon promotes hepatic glucose output by increasing glycogenolysis and gluconeogenesis, which leads to increased blood glucose levels (2).

Both insulin and glucagon were discovered for more than 100 years, and their functions were well studied in human and animal models (2, 3). Moreover, the sequences and functions of insulin and glucagon have been characterized from many non-model species of vertebrates. Although their gene number and sequence may differ among species, the functions of insulin and glucagon mature peptides are highly conserved during the evolution of vertebrates (4, 5). However, there was limited information about the molecular characteristics of insulin and glucagon in cetaceans. To our knowledge, insulin and glucagon sequences were only reported from pygmy sperm whale (*Kogia breviceps*) in cetaceans by our group (6). Interestingly, bottlenose dolphins (*Tursiops truncatus*), a cetacean species, have been reported to exhibit diabetes-like conditions and insulin resistance comparable to those observed in humans (7). Furthermore, they appear capable of switching this insulin-resistant state on and off in response to physiological demands (8). To explore both the universal and distinctive functions of these two hormones in cetaceans, it is of interest to further characterize their sequences, features, and functions in this group.

Pancreatic islets are highly organized micro-organs (9, 10) rather than unstructured endocrine cell clusters, which consist of several endocrine cell types, majorly including α cells, β cells, and δ cells (2). δ cells, accounting for approximately 5% of pancreatic islet cells, produce somatostatin and play a key role in modulating the secretory activity of neighboring α and β cells (11). Islet architecture is very important for proper function in response to nutritional stimuli (12). Mice islets have a β-cell-rich core which is surrounded by smaller numbers of α cells and δ cells, while in human islets the α cells, β cells, and δ cells maintained a more scattered organization throughout the islet (13, 14). The islet also displayed different patterns of endocrine cell arrangement in several cetacean species. In beluga whale (*Delphinapterus leucas*) islet, β cells were located centrally, and the α cells were located peripherally (15). The arrangements of α cell and β cell in bottlenose dolphin and pygmy sperm whale were found in such a way that β cells were clustered in the cords, while α cells were either dispersed or in the periphery (6, 16). However, several comparative studies indicated a striking plasticity of the islet architecture and cellular composition among different species and also within a single species under different physiological conditions (14). The known determinants of pancreatic islet architecture include cell adhesion molecules, cytoskeletal regulators, mesenchyme, vasculature, innervation, and β cell maturity (12).

As aquatic mammals, the finless porpoise (*Neophocaena* spp.) is a small-toothed whale, which belongs to phocoenids, mostly inhabiting shallow inshore waters (17). As their coastal habitat is heavily affected by the modern trawling industry and anthropogenic disturbance, they are particularly vulnerable to by-catch and stranding (18, 19) and have been listed as vulnerable or endangered species by the International Union for Conservation of Nature (IUCN) (20). Moreover, endocrine-disrupting chemicals (EDCs) in aquatic environments impair the development and health of finless porpoise (21, 22). In model organisms such as mice, the β cells and α cells of pancreatic islets have been established as targets of EDCs (23). However, definitive reports on the effects of EDCs on endocrine cells are lacking for the finless porpoise or any other cetacean species. Therefore, characterizing the insulin and glucagon genes in the finless porpoise, as well as the morphology of their corresponding endocrine cells, will enhance our understanding of cetacean physiology and contribute to conservation strategies for this species. In this study, we described the cloning of insulin and glucagon from finless porpoise. We also determined the islet distribution, architecture, and composition from different growth stages in this species.

## 2 Materials and methods

### 2.1 Animal and tissue collection

The five finless porpoise specimens investigated in this study were all collected from the coastal cities of Fujian Province, China, from 2019 to 2020 (**Table 1**). All specimens in this study were stranding carcass. They were transported to the Endangered Animal Center, Third Institute of Oceanography, Ministry of Natural Resources, Xiamen, China, immediately when found. The external morphology of the carcass was assessed before necropsy. Sample management and dissection were performed in compliance with the introductory guide for the anatomy of marine mammalian necropsy. The state of the finless porpoises was classified as fresh carcass (code 2) as described by Pugliares-Bonner et al. (24). Samples of several tissues (including skin, muscle, blubber, heart, liver, spleen, lung, kidney, and pancreas) were collected during necropsy and stored at −80 °C for further analysis. One set of pancreas was preserved in 1 mL RNA-later solution (Applied Biosystems, Warrington, UK) and stored at 4°C for 24 h, and then it was transferred to −80°C until RNA extraction. Several pieces of the pancreas were removed from the finless porpoises and fixed for 4 h in 4% paraformaldehyde–PBS at 4 °C. The pancreas was then equilibrated in 30% sucrose overnight at 4 °C until the tissue settled at the bottom. The pancreas was then placed in a cryomold containing optimal cutting temperature (OCT) compound and stored at −80 °C for further tissue analysis. The tissue collection protocol followed the study as previously reported (6, 25).

**Table 1:** Basic information on stranded finless porpoises.

Terms used in this study	Life history category	Gender	Body length (cm)	Body weight (kg)	Stranding location
Early fetus	Fetus	Male	38	0.85	Fuzhou, Fujian, China
Late fetus	Fetus	Male	79	4.55	Ningde, Fujian, China
Male	Sub-adult	Male	114	21.94	Pingtan, Fujian, China
Female	Sub-adult	Female	113	18.90	Pingtan, Fujian, China
Pregnant female	Pregnant adult	Female	138	59.00	Ningde, Fujian, China

### 2.2 Molecular cloning, structural analysis, synteny map, and phylogenetic analysis of insulin and glucagon genes

Genomic DNA was extracted from muscle tissues using the DNeasy Blood and Tissue Kit (Qiagen, Hilden, Germany), following the manufacturer’s instructions. For species identification, a partial fragment of the D_loop region was amplified and sequenced using primers Ce-CRF: 5′-GAATTCCCCGGTCTTGTAAACC-3′ and Ce-CRR: 5′-TCTCGAGATTTTCAGTGTCTTGCTTT-3′ (26). Total RNA was isolated from pancreatic tissues using TRIzol reagent (Thermo Fisher Scientific, Waltham, MA, USA) according to the manufacturer’s instruction. The first-strand cDNA was synthesized with reverse transcription system (TaKaRa) according to the manufacturer’s protocol. cDNA was first used to clone the beta-actin gene to verify its quality before subsequent gene cloning. Two pairs of primers were designed based on the CDS (coding sequence) of insulin (cDNA-INS-F1: 5′-ATGGTCCTGTGGATGCGC-3′, cDNA-INS-R1: 5′-AGTTGCAGTAGTTCTCCAGCTG-3′) and glucagon (cDNA-GCG-F1: 5′-ATGAAAAGCATTTACTTTGTGGCTG-3′, cDNA-GCG-R1: 5′-TTACTTCCTGTCAGTAACTTTTGTC-3′). The PCR was performed using TaKaRa LA Taq with GC Buffer system (TaKaRa Bio Inc.) in a Veriti 96-well thermal cycler (Applied Biosystem, CA, USA). The PCR products were estimated by using 1% agarose gel electrophoresis and sequenced in both directions at the Majorbio Sequencing (Shanghai, China).

The cetacean insulin and glucagon amino acid sequences for alignment or analysis were obtained from the NCBI database, including bottlenose dolphin (*Tursiops truncatus*), Pacific white-sided dolphin (*Lagenorhynchus obliquidens*), killer whale (*Orcinus orca*), beluga whale (*Delphinapterus leucas*), Baiji (*Lipotes vexillifer*), pygmy sperm whale (*Kogia breviceps*), sperm whale (*Physeter catodon*), and minke whale (*Balaenoptera acutorostrata scammoni*). Sequence alignment analysis was performed using GeneDoc V 2.7.0.

Phylogenetic analysis was done using full-length amino acid sequences including 32 mammals (*Neophocaena phocaenoides*, *Delphinapterus leucas*, *Orcinus orca*, *Tursiops truncates*, *Lagenorhynchus obliquidens*, *Lipotes vexillifer*, *Physeter catodon*, *Kogia breviceps*, *Balaenoptera acutorostrata*, *Ovis aries*, *Bos taurus*, *Sus scrofa*, *Equus caballus*, *Equus przewalskii*, *Equus asinus*, *Canis lupus familiaris*, *Vulpes vulpes*, *Phoca vituline*, *Eumetopias jubatus*, *Tupaia chinensis*, *Pongo abelii*, *Gorilla gorilla*, *Homo sapiens*, *Echinops telfairi*, *Loxodonta africana*, *Trichechus manatus latirostris*, *Oryctolagus cuniculus*, *Ochotona princeps*, *Peromyscus leucopus*, *Rattus norvegicus*, *Mus musculus*, and *Ornithorhynchus anatinus*), one Aves (*Gallus gallus*), one Reptilia (*Alligator sinensis*), one Amphibia (*Xenopus tropicalis*), and one Actinopterygii (*Danio rerio*) by maximum likelihood method using the General Time Reversible model by MEGA 7.0 with 1,000 bootstraps (The Biodesign Institute, Tempe, AZ, USA).

Synteny data among different species were obtained from the Genome Data Viewer (https://www.ncbi.nlm.nih.gov/gdv). The neighboring genes for *insulin* and *glucagon* in these species were based on the following genome assemblies: finless porpoise (V1.1), human (GRCh38.p14), pig (Sscrofa11.1), mouse (GRCm39), chicken (GRCg7b), frog (UCB_Xtro_10.0), and zebrafish (GRCz12tu).

### 2.3 Hematoxylin and eosin staining

OCT-frozen samples from finless porpoise were cut into 9-μm-thick sections, and the slices were pretreated with 4% paraformaldehyde–PBS. Next, the slices were stained with hematoxylin for 1 min, differentiated with 1% hydrochloric acid for 30 s, counterstained with eosin for 30 s, rinsed with running water for 5 min, then dehydrated with different concentrations of ethanol (70%, 80%, and 95% ethanol and absolute ethanol), transparentized with xylene, and finally mounted in neutral gum.

### 2.4 Immunofluorescence

OCT-frozen pancreas of finless porpoises at different developmental stages were cut into 9-μm-thick sections. For immunofluorescence quantification of islet area, between the sections for staining, we discarded 10 sections to prevent repeated staining of the same cells. Guinea pig anti-insulin antibody (DAKO, A0564) was used to stain islet β cells, and mouse anti-glucagon antibody (Sigma, G2654) was used to stain islet α cells, followed by secondary antibodies which were Alexa Fluor-conjugated goat antibodies (Thermo Fisher Scientific). Confocal microscopy and laser scanning microscope software (Leica TCS SP8 STED) were used to measure the colocalization and capture images.

### 2.5 Immunohistochemistry

OCT-frozen samples from finless porpoises at different developmental stages were cut into 9-μm-thick sections. When we performed the immunochemistry for islet area quantification, we cut one slice for staining, then cut 10 slices and discard them, and then cut another slice for another round of staining to avoid performing staining on the same cell. The slices were pretreated with 4% paraformaldehyde–PBS, 1% Triton X-100 in PBS, 3% hydrogen peroxide, 0.1 M glycine, and blocking buffer (5% FBS and 0.1% Tween-20 in PBS) successively. The pancreas sections were stained using mouse anti-insulin antibody (Beyotime, AF0204) for β cells and mouse anti-glucagon antibody (Sigma, G2654) for α cells, and then the sections of the abovementioned treatment were incubated with horseradish peroxidase-conjugated secondary antibody (ZSGB-BIO, PV-9000) for 1 h. After a positive reaction was observed with diaminobenzidine (ZSGB-BIO, ZLI-9018), it was immediately terminated with water. After that, the sections were counterstained with hematoxylin, differentiated with 1% hydrochloric acid, and then dehydrated and sealed.

### 2.6 Quantification of islet area

The pancreatic regions from different sections were randomly selected and imaged at ×100 magnification. ImageJ software (NIH, Bethesda, MD, USA) was used to quantitatively analyze the areas stained by insulin and/or glucagon in the pancreas by adjusting the threshold and selecting the effective range. At least five images from three different sections were quantified. The data were expressed as mean ± S.E.M., which represented the average of glucagon-staining section (α cell areas), insulin-staining section (β cell areas), or insulin-plus-glucagon-staining sections (α cell + β cell areas) from the imaged pancreatic area.

### 2.7 Quantification of the proportion of endocrine cells

In the immunofluorescence staining sections of the pancreas of finless porpoises at different developmental stages, the pancreatic regions were randomly selected, and the number of α cells and β cells, respectively, with different fluorescence colors within each islet was determined manually. The percentage of α cells and β cells, respectively, was calculated as the number of α cells or β cells compared with the total number of α cells plus β cells. At least seven islets from three different sections were quantified.

### 2.8 Transmission electron microscopy imaging of endocrine cells

Finless porpoise pancreatic tissue was cut into long strips with a diameter of 1 mm and fixed with 2.5% glutaraldehyde solution in a refrigerator at 4 °C for 12 h. After fixation, the tissues were rinsed in 0.1 M phosphate buffer containing Na_2_HPO_4_ and NaH_2_PO_4_, then dehydrated, and embedded in resin. The images were taken with a Hitachi ht-7800 microscope.

### 2.9 Statistical analysis

Data were presented as means and S.E.M. All analyses were performed using GraphPad Prism 8 software (Graph-Pad Software v8.3.0, La Jolla, CA, USA).

## 3 Results

### 3.1 Characterization and molecular identification of the specimen

A total of five specimens, including two fetuses (one early fetus and one late fetus), one sub-adult male (referred to as male), one sub-adult female (referred to as female), and one pregnant specimen (referred to as pregnant female), were collected in this study. The biological features of the samples, including sex and size, are presented in **Table 1**. The age of each porpoise was estimated based on the sex and body length (27, 28). In order to further identify the species, we successfully amplified an 895-bp mitochondrial D-loop-region fragment from all specimens. Significant sequence alignments of the D-loop regions from the five specimens, identified using the Basic Local Alignment Search Tool (BLAST search), showed 100% sequence identity with *N. phocaenoides* sequences (**Supplementary Table 1**).

### 3.2 Identification and characterization of finless porpoise *insulin* and *glucagon* genes

**Figure 1:**
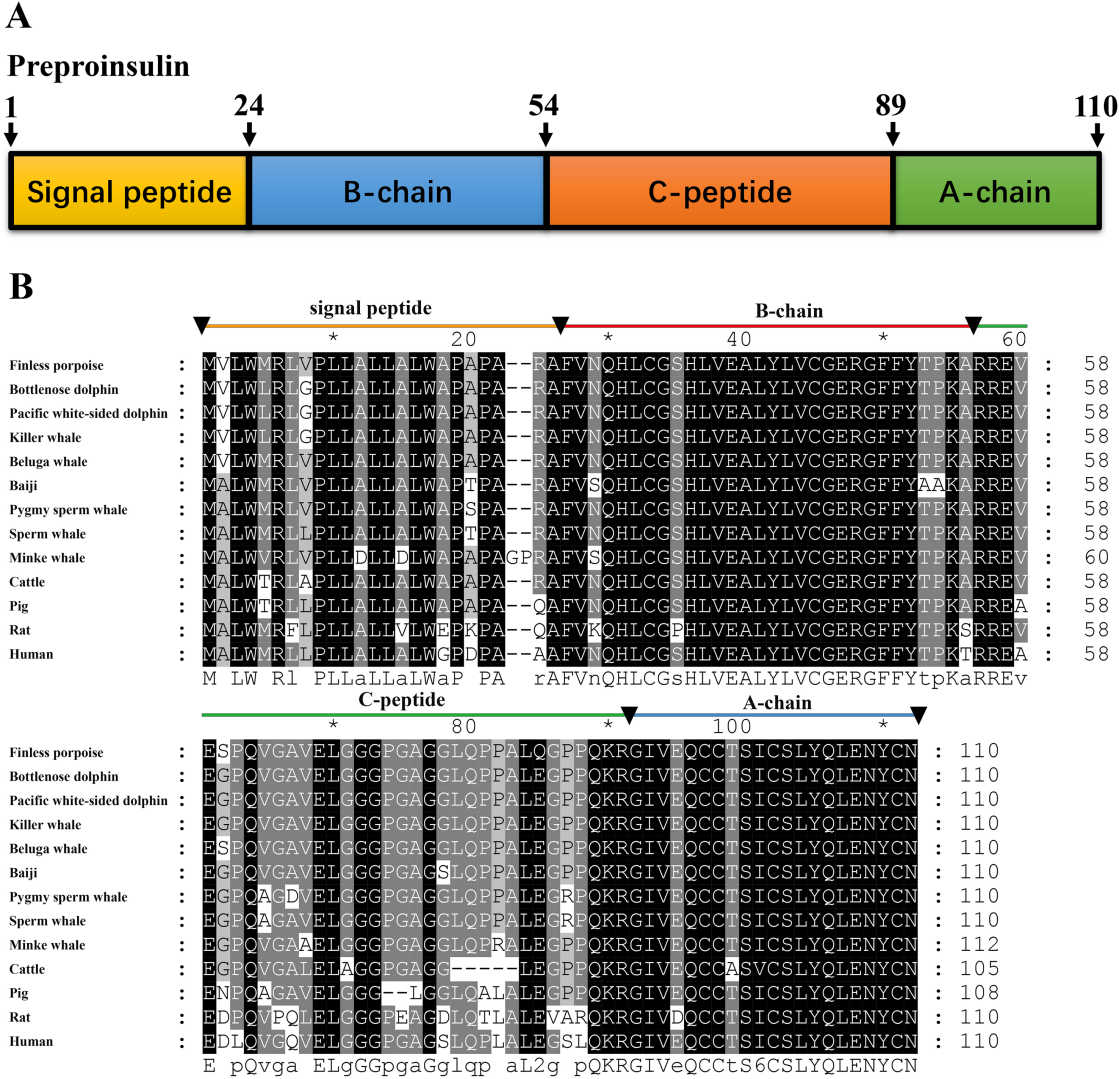
Cloning of *preproinsulin* gene from finless porpoise. **(A)** Domain structure of finless porpoise insulin, showing the signal peptide (residues 1–24), B-chain (25–54), C-peptide (55–89), and A-chain (90–110). **(B)** Sequence alignment of finless porpoise preproinsulin with orthologs from other vertebrates, generated using Genedoc with the Clustal method. Conserved and identical residues are shaded.

For *insulin* gene, the coding sequence (CDS) was amplified by RT-PCR using mRNA isolated from the pancreas of the porpoise. The full-length CDS of *insulin* is 333 bp, encoding a 110-amino-acid preproinsulin protein. The nucleotide and deduced amino acid sequence have been deposited in GenBank (accession no. PV926279). The preproinsulin contained a signal peptide (residues 1–24), B chain (residues 25–54), C-peptide (residues 55–89), and A chain (residues 90–110) (**Figure 1A**). The mature finless porpoise insulin peptide, A-chain and B-chain, was highly conserved, showing 100% identity with many cetacean preproinsulin orthologs, except for Baiji and minke whale. However, the signal peptide and the C-peptide were more variable among different mammalian preproinsulin orthologs (**Figure 1B**).

**Figure 2:**
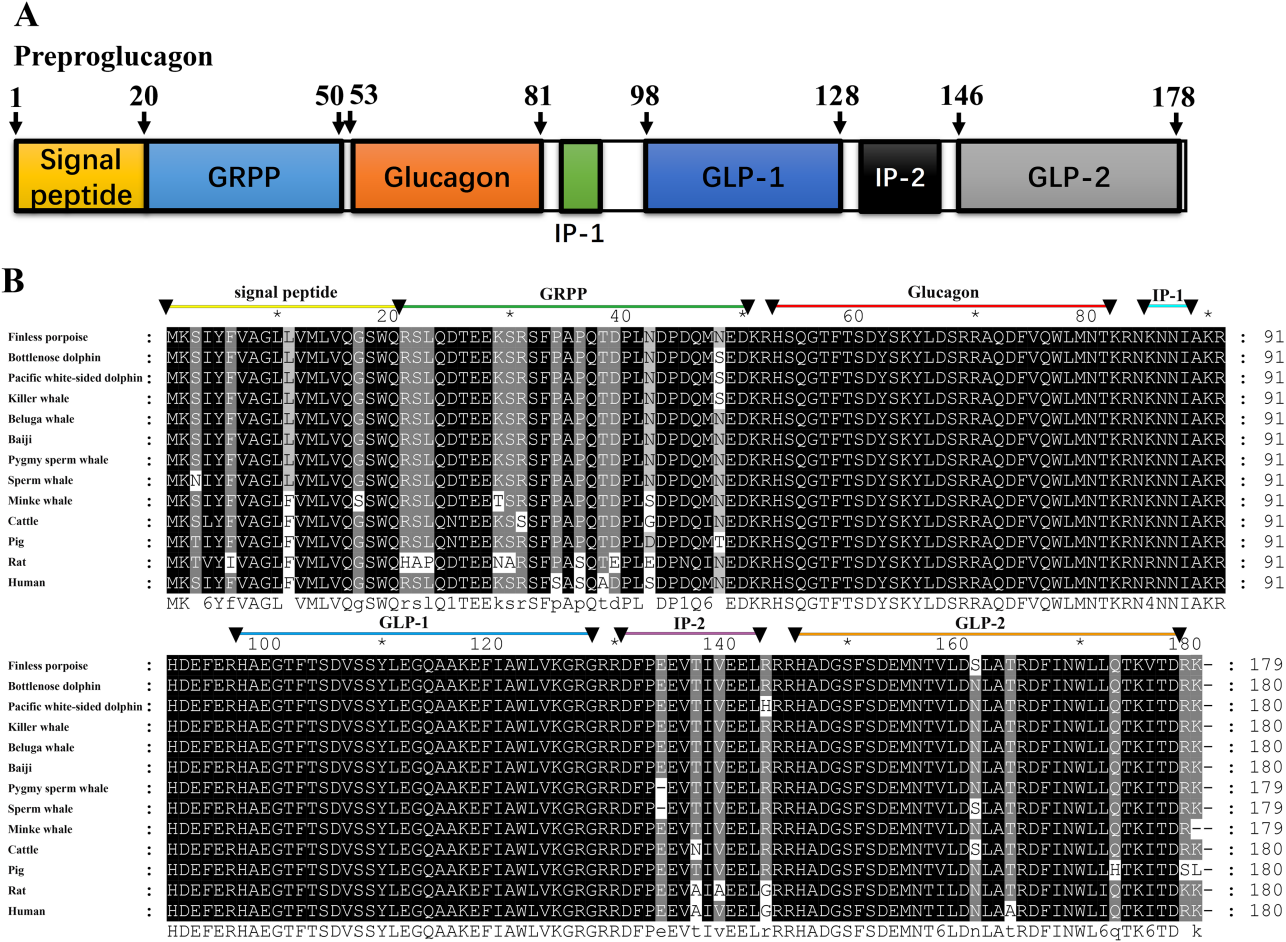
Cloning of *pregproglucagon* gene from finless porpoise. **(A)** Domain structure of finless porpoise glucagon, showing the signal peptide (residues 1–20), GRPP (21–50), glucagon (53–81), GLP-1 (98–128), and GLP-2 (146–178). **(B)** Sequence alignment of finless porpoise preproglucagon with orthologs from other vertebrates, generated using Genedoc with the Clustal method. Conserved and identical residues are shaded.

The CDS of *glucagon* was also amplified by using RT-PCR, which was 543 bp and encoded the full-length preproglucagon of 180 aa (**Figure 2A**). The nucleotide and deduced amino acid sequence have been deposited in GenBank (accession no. PV926280). The sequences of mature glucagon peptide (residues 53–81) and GLP-1 (residues 98–128) were highly conserved, showing 100% identity among mammalian orthologs (**Figure 2B**).

### 3.3 Phylogenetic analysis and synteny analysis of finless porpoise insulin and glucagon

We next performed a phylogenetic analysis using the preproinsulin and preproglucagon sequences of 36 vertebrates obtained from GenBank. The phylogenetic tree constructed using the maximum likelihood method revealed that both finless porpoise preproinsulin and preproglucagon clustered within the predicted cetacean preproinsulin or preproglucagon, with the closest match to *Delphinapterus leucas* with high bootstrap values, suggesting that the two genes obtained in this study were indeed the orthologs of cetacean preproinsulin and preproglucagon (**Supplementary Figure 1**).

**Figure 3:**
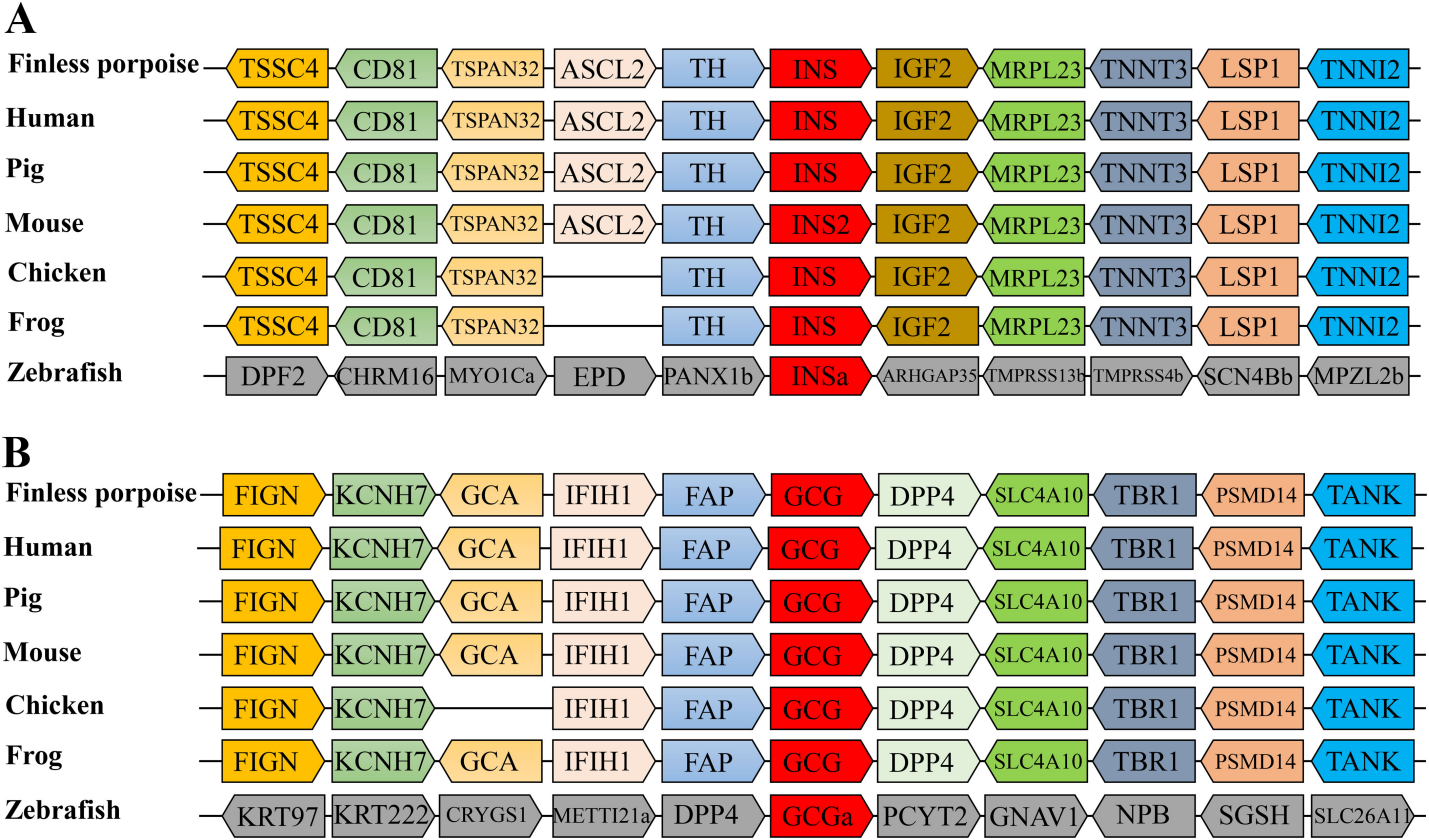
Synteny map of the genomic segment showing the insulin and glucagon residues in seven vertebrate genomes. **(A)** Synteny map of the insulin genes: TSSC4 (tumor-suppressing subtransferable candidate 4), CD81 (CD81 molecule), TSPAN32 (tetraspanin 32), ASCL2 (achaete-scute family BHLH transcription factor 2), TH (tyrosine hydroxylase), Ins (insulin), MRPL23 (mitochondrial ribosomal protein L23), TNNT3 (troponin T3, fast skeletal type), LSP1 (lymphocyte-specific protein 1), and TNNI2 (troponin I2, fast skeletal type). **(B)** Synteny map of the glucagon genes: FIGN (Fidgetin-like 2), KCNH7 (potassium voltage-gated channel subfamily H member 7), GCA (grancalcin), IFIH1 (interferon induced with helicase C domain 1), FAP (fibroblast activation protein alpha), GCG (glucagon), DPP4 (dipeptidyl peptidase 4), SLC4A10 (solute carrier family 4 member 10), TBR1 (T-box, brain 1), PSMD14 (proteasome 26S subunit, non-ATPase 14), and TANK (TRAF family member associated NFKB activator). Genes are represented by arrows with different colors. The transcription direction is indicated by the arrow.

To explore the possible synteny relationships among different vertebrate insulin genes, we analyzed genes surrounding the insulin locus and glucagon locus, covering five other genes on both sides of the insulin and glucagon genes, respectively. For most species, the genes located upstream of the insulin locus included TH, ASCL2, TSPAN32, CD81, and TSSC4. In the proximity downstream of the insulin genes, there were IGF2, MRPL23, TNNT3, LSP1, and TNNI2 (**Figure 3A**). The ASCL2 gene in chicken and frog has not been detected at the insulin locus. For the glucagon locus, the genes located upstream of the glucagon locus include FAP, IFIH1, GCA, KCNH7, and FIGN. In the proximity downstream of the GCG genes, there were DPP4, SLC4A10, TBR1, PSMD14, and TANK (**Figure 3B**). Overall, the order of arrangement of these genes among different species’ genomes was highly conserved surrounding the insulin locus and the glucagon locus except for zebrafish. These highly conserved synteny relationships among different vertebrates provided further evidence that the genes we cloned are indeed finless porpoise insulin and glucagon genes.

### 3.4 Distribution of islets in finless porpoise pancreas

**Figure 4:**
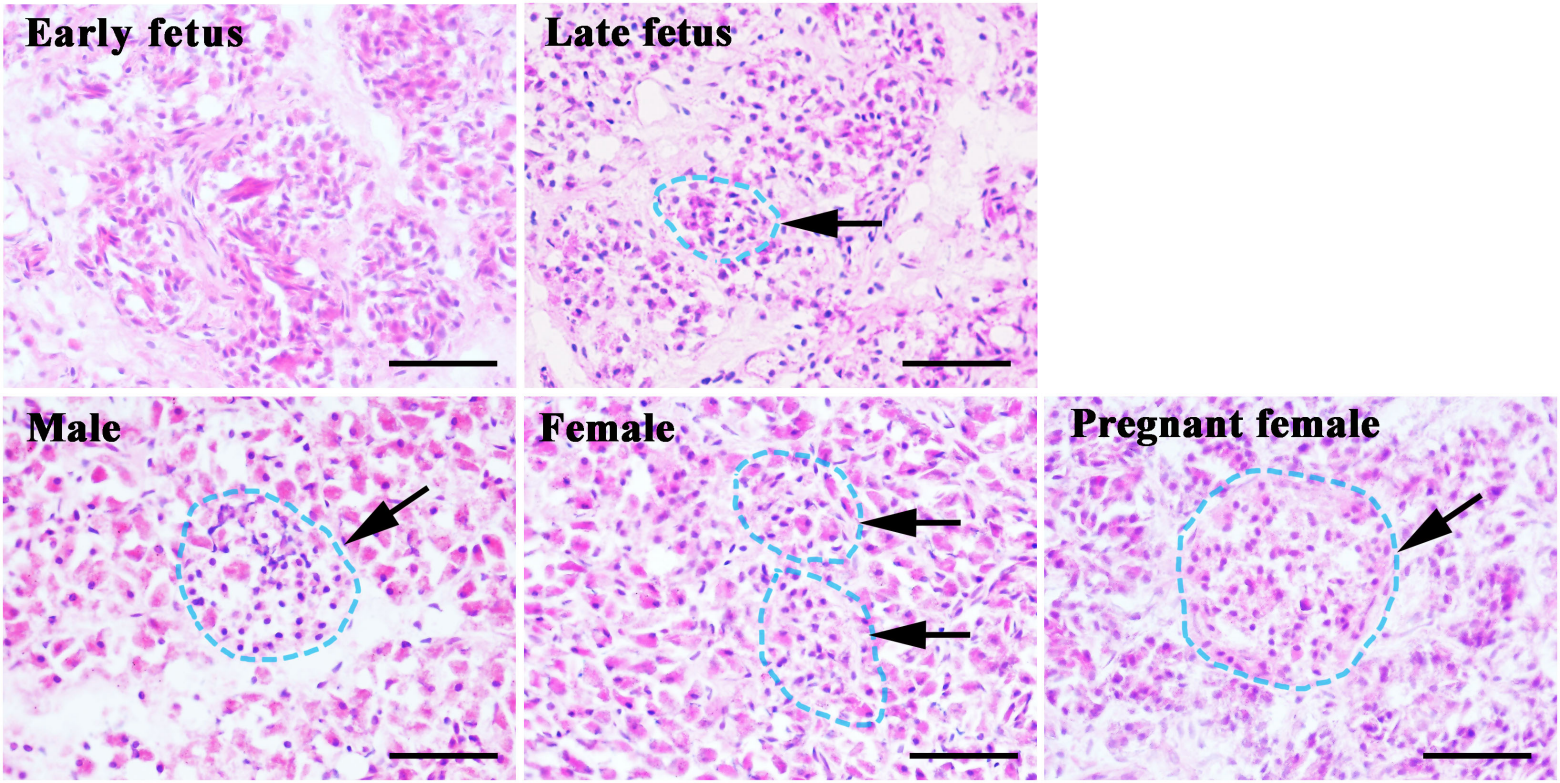
Distribution of islets in different growth stages of finless porpoise. The pancreas of finless porpoise were stained with hematoxylin and eosin. The different stages of finless porpoise were as indicated. Arrows and blue dotted circles indicate the islet of each species or different stages of finless porpoise. The scale bar indicates 50 μm.

To identify the islet area in different growth stages or genders in finless porpoises, we performed hematoxylin and eosin staining (H&E) on the finless porpoise pancreas sections. As shown in **Figure 4**, the boundary of the acinar cell and islet was obscure in finless porpoises (**Figure 4**). In addition, no islet shape was seen in the early fetus, and a few islets were detected in the late fetus. However, it was easy to find islets in the sub-adult male, sub-adult female, and pregnant female finless porpoise pancreas (**Figure 4**).

**Figure 5:**
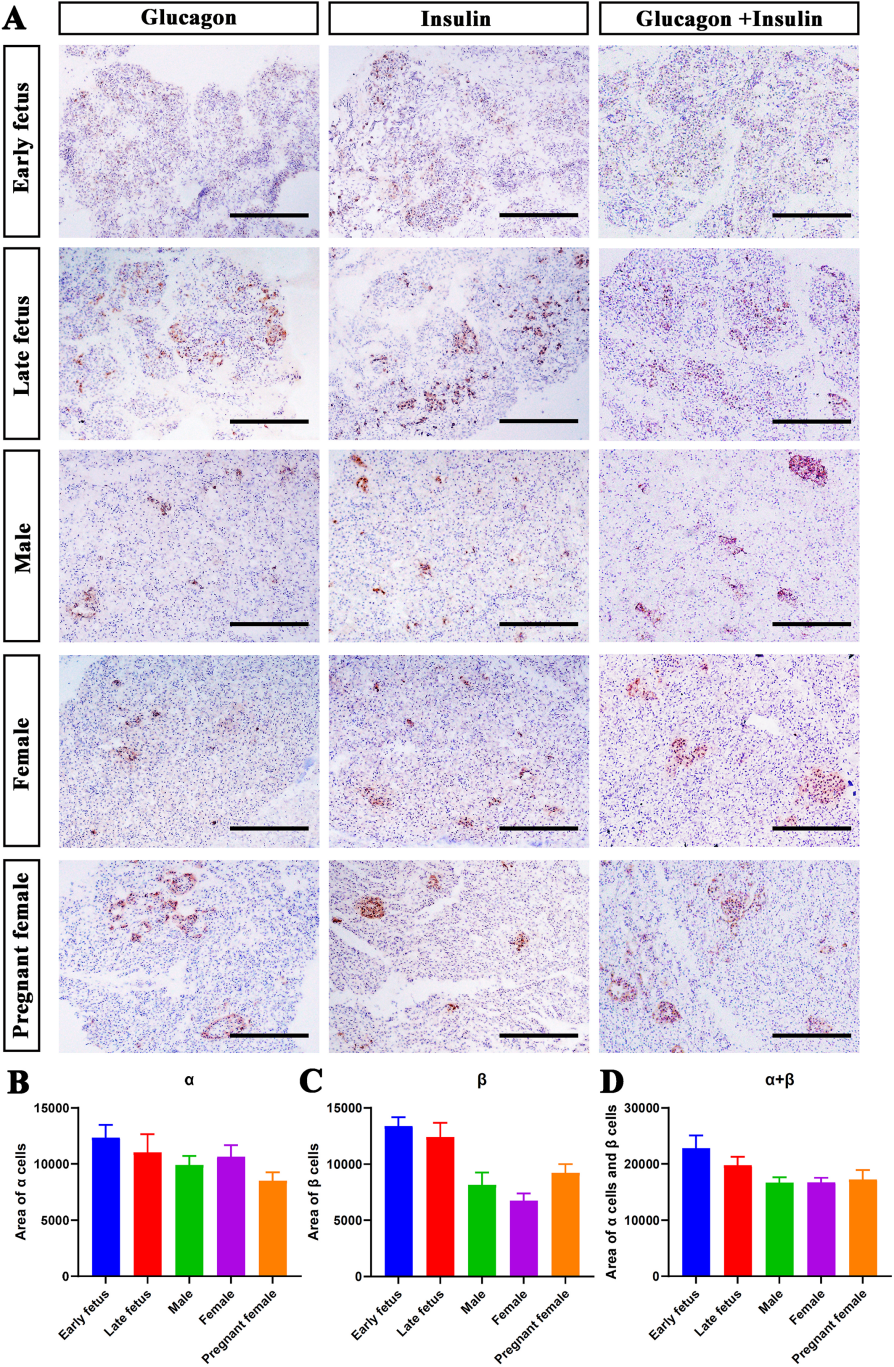
Distribution of α and β cells in finless porpoises at different growth stages. **(A)** Representative immunohistochemical images of the finless porpoise pancreas at different growth stages. α cells were stained with an anti-glucagon antibody and β cells with an anti-insulin antibody. The scale bar indicates 200 µm. **(B–D)** Quantification of the areas of α cells, β cells, and α cell plus β cells in finless porpoises at different growth stages.

We further performed immunohistochemistry staining with insulin, glucagon, or insulin plus glucagon antibodies. As shown in **Figure 5**, the α and β cells did not form the islets in the early fetal stage of finless porpoise, which scattered throughout the pancreas (**Figure 5A**, the top panel). At the age of late fetus, the islet started to form clusters, albeit still quite a lot of α and β cells without islet outlines were scattered in the pancreas (**Figure 5A**, the second panel). In the sub-adult and pregnant stages, most of the α and β cells clustered in the islets, and the islets were randomly distributed in the pancreas with various sizes and shapes (**Figure 5A**, third panel to fifth panel). We also quantified the areas of α cells, β cells, and α cells plus β cells from different stages or gender sections. All of the areas of α cells, β cells, and α cells plus β cells per section had the trend to decrease (**Figures 5B–D**). Interestingly, the area of β cells was increased in pregnant female compared with non-pregnant female porpoises (**Figures 5B–D**).

### 3.5 Architecture of finless porpoise islet

**Figure 6:**
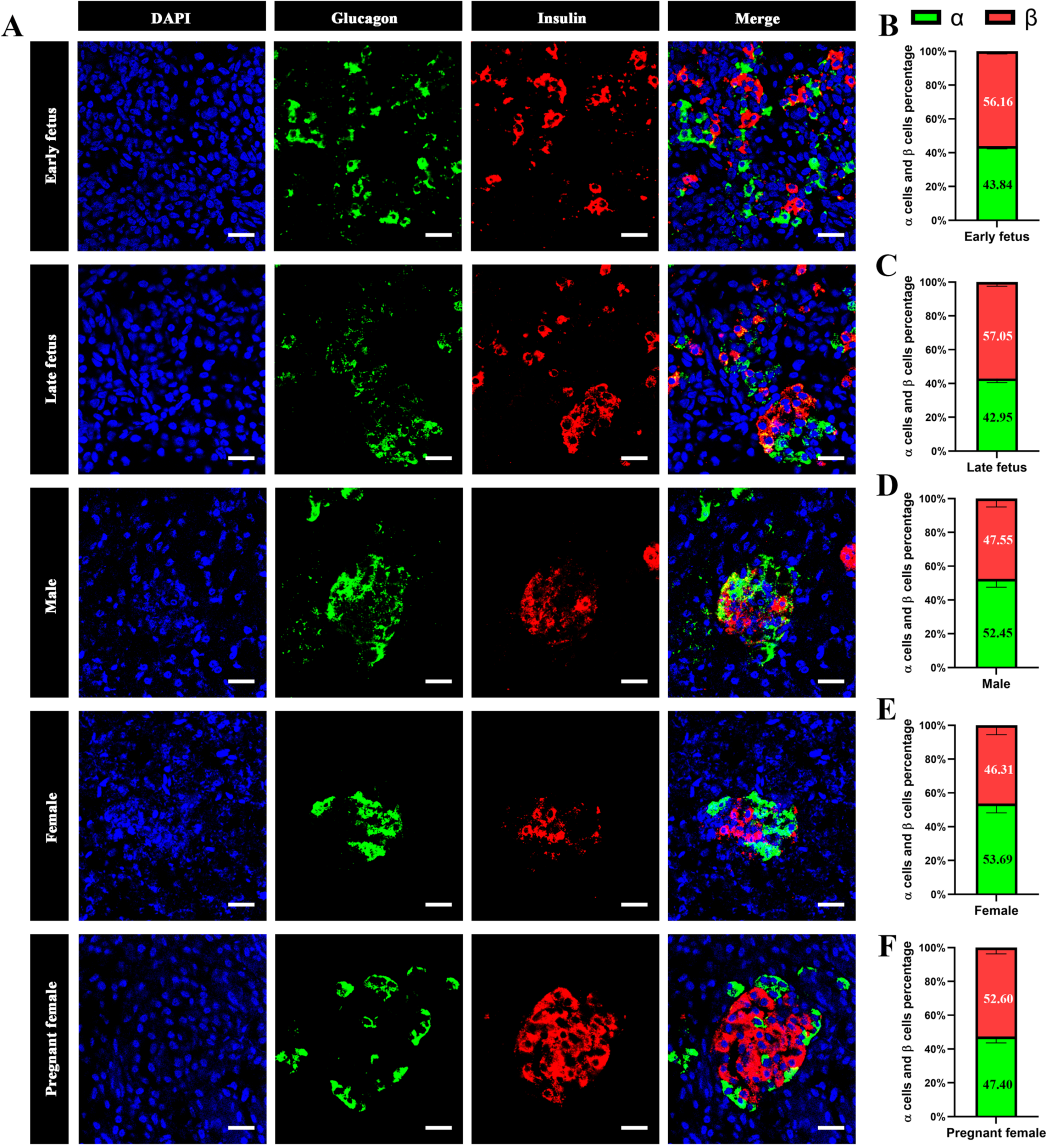
Architecture of α cells and β cells in finless porpoise islet. **(A)** Representative confocal images of α and β cells in the pancreas of finless porpoises at different growth stages, obtained by immunofluorescence staining. The nuclei are stained with DAPI (blue), α cells are labeled with an anti-glucagon antibody (green), and β cells are labeled with an anti-insulin antibody (red). Images were acquired using a ×63 objective lens. The scale bars indicate 20 µm. **(B–F)** Bar charts showing the proportions of α and β cells within the islets across different growth stages and sexes.

**Figure 7:**
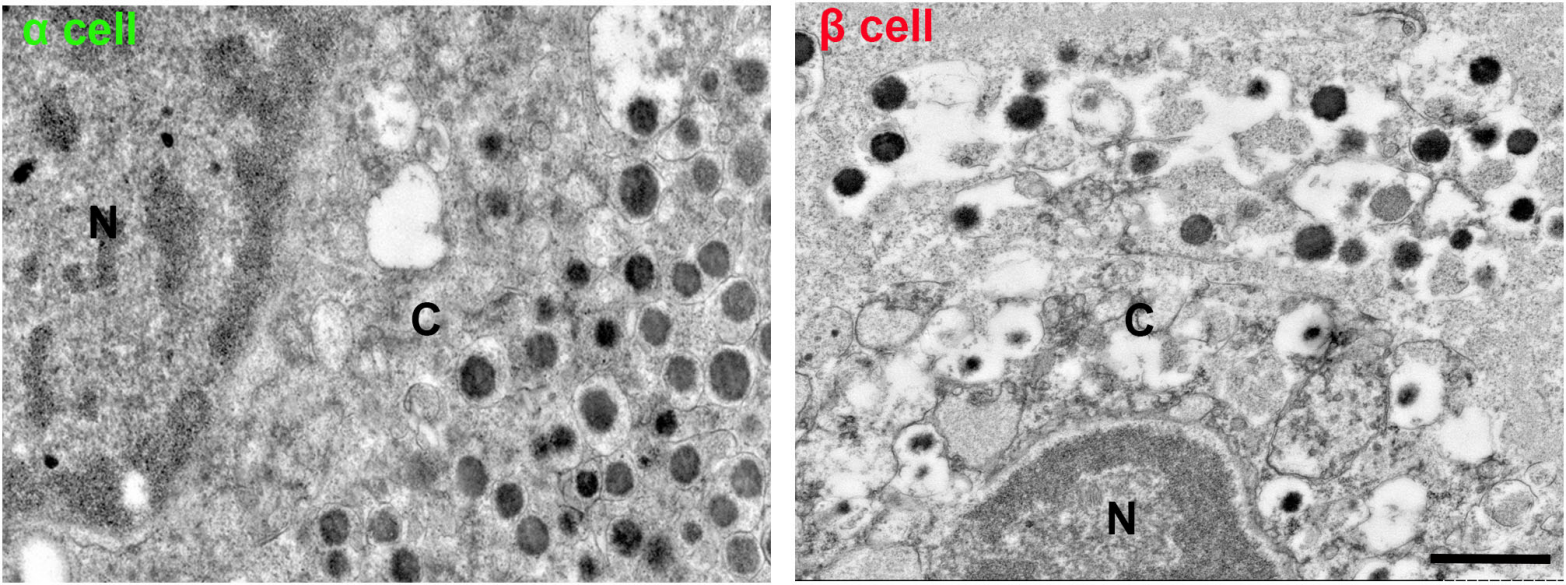
Transmission electron microscopy (TEM) imaging of α and β cells in adult finless porpoise. α cells were identified by the presence of round, dense-core vesicles surrounded by a small halo. In contrast, β cells were characterized by immature secretory granules with a light core as well as mature secretory granules containing a dense core and a prominent halo. N, nucleus; C, cytosol. Scale bar = 1 µm.

To explore the architecture of the islet in different stages or gender of finless porpoise, we then performed multicolor immunostaining using antibodies against glucagon and insulin. As shown in **Figure 6A**, in the early fetus stage, the α and β cells were randomly distributed in the pancreas without an islet outline. In the age of late fetus, the α and β cells started to form islets. In the sub-adult stage, the α and β cells were randomly distributed throughout the islet without principle. In the pregnant stage, the islets displayed β cells in the islet core, and most α cells were in the islet periphery, with a few scattered throughout the islets. The percentage of β cells was higher than that of α cells in early fetus, late fetus, and pregnant stages (**Figures 6B–F**). We further conducted a transmission electron microscopy analysis of the pancreas of pregnant female finless porpoises. The ultrastructural imaging revealed that it had numerous glucagon granules in α cells and lots of insulin granules within their β cells. Moreover, the shape of the granules was similar to those of other mammalian species. The α cells showed round dense core vesicles with a small halo, while the β cells displayed with a dense core and a prominent halo (**Figure 7**).

## 4 Discussion

In this study, we cloned and characterized both *preproinsulin* and *preproglucagon* genes from finless porpoises, determined their primary structures, and demonstrated their conserved synteny among mammals. The finless porpoise preproinsulin protein exhibited a similar domain arrangement with other known preproinsulins, and the mature insulin peptide (B-chain and A-chain) was 100% identical in most cetacean species, except for Baiji and minke whale, which belong to different families or suborders (29). However, it was also 100% identical to that of the pig (*Sus scrofa*), belonging to the Artiodactyla group (30), and had only one amino acid difference with the human insulin sequence (**Figure 1A**). Similarly, the preproglucagon protein exhibited strong conservation in primary structure and synteny. The encoded protein shared a high sequence similarity with other mammals, particularly in the mature glucagon and GLP-1 peptide regions, showing 100% identity across all examined species, including cetaceans, humans, mice, pigs, and cattle (**Figure 2**). These findings suggest that the functional roles of insulin and glucagon are likely conserved in finless porpoises.

We further combined H&E staining, immunohistochemistry, and immunofluorescence approaches to investigate the islet distribution and architecture in finless porpoises from different growth stages. From the morphological standpoint, the shapes of finless porpoise islets were irregular and scattered throughout the pancreatic lobules (**Figures 4**, **5**), which was similar to our recent finding in the pygmy sperm whale (6). However, various islet shapes of different marine mammals have been reported, including elongated shapes, ovoid shapes, and irregular shapes (6, 16, 31).

In addition, the pancreatic islet architecture and endocrine cell composition changed across different growth phases in finless porpoises. The α and β cells did not form the islets in the early fetus stage of finless porpoise; however, the islets started to form in the late fetus stage, albeit still quite a lot of α and β cells without islet outlines were scattered in the pancreas (**Figures 5**, **6A**). In the sub-adult stage, the α and β cells formed the shape of the islets and randomly distributed throughout the pancreas (**Figure 5**). However, the α cells and the β cells appeared to be randomly distributed inside the islets, suggesting that islet maturation remained incomplete (**Figure 6A**). In the pregnant stage, the islets displayed the β cells in the islet core and most α cells in the islet periphery, with a few scattered throughout the islets (**Figure 6A**). The islet architecture of pregnant stage represented a mature stage, which was highly similar to the islet architecture of bottlenose dolphin, pygmy sperm whale, and beluga whale (6, 15, 16). However, it was different with mice and human. In mice, the β cells highly concentrated in the islet core, and the α cells surrounded at the periphery, while in humans, the α cells and the β cells often intermingled rather than strictly segregated (14).

The dynamic changes of islet architecture in different stages of finless porpoises were highly similar to those of other mammalian species, like mouse and pig (32, 33). The α cell and the β cell composition, respectively, of finless porpoises were very close (**Figure 6**). Interestingly, the percentage of β cells was higher in the fetal and pregnant stages (**Figures 6B–F**), and the area and composition of the β cells were increased in pregnant female compared with non-pregnant female porpoises (**Figures 5C**, **6F**). Similar to our observation, the fraction of the β cell area is higher in infancy and gradually declines thereafter to adulthood in humans (34). It has also been reported in mice that the physiological states, such as pregnancy, can affect the islet composition and architecture, with increased β cell mass observed in response to elevated metabolic demand (13, 35, 36).

Although pancreatic islets and their key endocrine factors are known to regulate glucose metabolism in humans and other organisms, their roles in cetaceans remain poorly understood. During the transition from land to water, cetaceans adapted to a diet composed almost exclusively of protein and lipids, with negligible carbohydrates. Under this nutritional framework, the physiological roles of insulin and glucagon in glucose homeostasis may diverge from those observed in terrestrial mammals. It is well documented that amino acids (e.g., arginine) potently stimulate both insulin and glucagon secretion in humans and rodents (37, 38). Consequently, in the carbohydrate-limited environment characteristic of cetaceans, dietary amino acids may act as particularly potent secretagogues for these pancreatic hormones.

However, this study has some limitations. First, because it is very difficult to obtain multiple freshly stranded animals at specific developmental stages, only one animal was available for each stage of the finless porpoise examined. Second, whether the differences in the amino acid sequences of proinsulin and proglucagon between humans and dolphins affect their functional properties requires further investigation.

In summary, we cloned and characterized insulin and glucagon genes from finless porpoises, which revealed a strong conservation in primary structure and synteny with other mammals. We also provided comprehensive information on islet distribution, architecture, and composition from different growth stages and genders in cetaceans for the first time. These findings provided new insights into the structural conservation of insulin and glucagon genes as well as the developmental distribution of endocrine cells in cetaceans. The high sequence conservation of these genes, coupled with the preserved islet localization of insulin and glucagon, indicates that despite major lifestyle changes and nutritional adaptations during the evolution from terrestrial to fully aquatic life, cetaceans have retained the physiological functions of these critical hormones. Moreover, endocrine-disrupting chemicals (EDCs) in aquatic environments impair cetacean health. Studies on the key endocrine factors and their morphological characterization in finless porpoises will support research on their conservation. Future studies will investigate how these endocrine factors regulate glucose homeostasis and their interactions with EDCs in cetaceans.

## Data Availability

The datasets presented in this study can be found in online repositories. The names of the repository/repositories and accession number(s) can be found in the article/**Supplementary Material**.
