# Supplementary material for: Identification of insulin and glucagon genes in the finless porpoise and the developmental distribution of their producing endocrine cells
Source: Front Endocrinol (Lausanne). 2026 Apr 30;17:1777351. doi: 10.3389/fendo.2026.1777351 (PMC13171375; doi:10.3389/fendo.2026.1777351)
Supplement: Supplementary file 1 [file DataSheet1.docx]

**Supplemental Figures and legend**

**Supplemental Table.1 Results from GenBank BLAST searches of the D-loop sequences obtained from finless porpoise specimens**

| **Name used in this study** | **GenBank NB** | **Closest matches with GenBank sequence** |
| --- | --- | --- |
|  |  | **Species (GenBank reference) and Identities (percentages)** |
| Pregnant female | MZ363477 | *N. phocaenoides*（AF289329）895/895(100%) |
| Female | MZ363478 | *N. phocaenoide*s（[AF289287](https://www.ncbi.nlm.nih.gov/nucleotide/AF289287.1?report=genbank&log$=nuclalign&blast_rank=1&RID=C07WA7DT016)）895/895(100%) |
| Male | MZ363479 | *N. phocaenoides*（[KX650871](https://www.ncbi.nlm.nih.gov/nucleotide/KX650871.1?report=genbank&log$=nuclalign&blast_rank=1&RID=C07ZHX70013)）895/895(100%) |
| Early Fetus | MZ363480 | *N. phocaenoides*（AF289287）895/895(100%) |
| Late Fetus | MZ363481 | *N. phocaenoides*（AF289329）895/895(100%) |


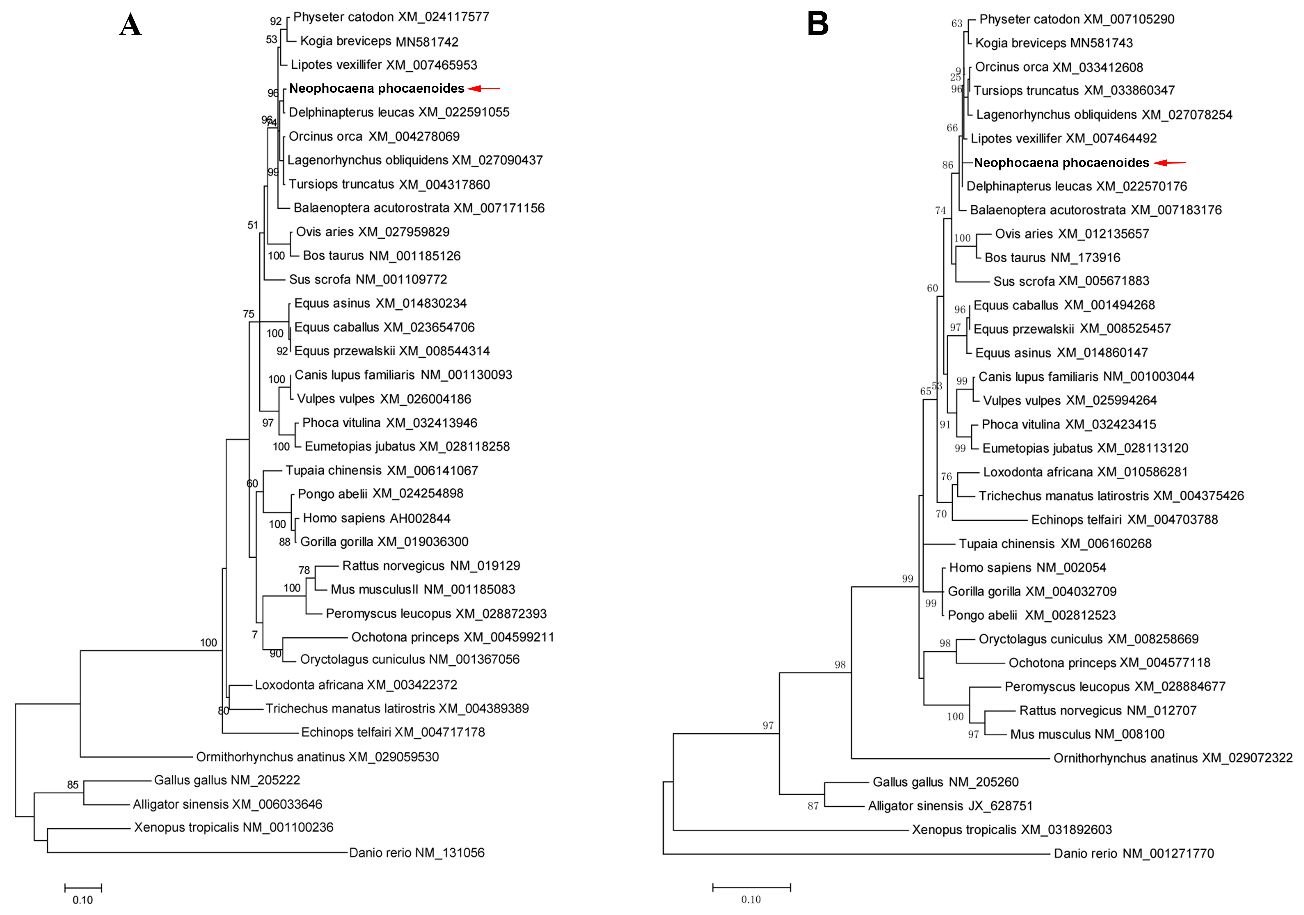


**Supplemental Figure 1.** **Phylogenetic analysis of the insulin and glucagon gene family**. Full-length sequences of preproinsulin (**A**) and preproglucagon (**B**) from difference species were analyzed using the Maximum likelihood method. Numbers on nodes represent frequency with which this node is recovered per 100 bootstrap replications in a total of 1000.
